# *MoHG1* Regulates Fungal Development and Virulence in *Magnaporthe oryzae*

**DOI:** 10.3390/jof10090663

**Published:** 2024-09-21

**Authors:** Xin Pu, Aijia Lin, Chun Wang, Sauban Musa Jibril, Xinyun Yang, Kexin Yang, Chengyun Li, Yi Wang

**Affiliations:** 1 State Key Laboratory for Conservation and Utilization of Bio-Resources in Yunnan, Yunnan Agricultural University, Kunming 650201, China; puxin_1998@163.com (X.P.); laj7411@126.com (A.L.); chunwang775@gmail.com (C.W.); saubanzango@gmail.com (S.M.J.); yang-xinyun2001@163.com (X.Y.); 18468006252@163.com (K.Y.); 2 Yunnan-CABI Joint Laboratory for Integrated Prevention and Control of Transboundary Pests, Yunnan Agricultural University, Kunming 650201, China

**Keywords:** *MoHG1*, cell wall integrity, chitin, hexokinase, rice blast

## Abstract

*Magnaporthe oryzae* causes rice blast disease, which threatens global rice production. The interaction between *M. oryzae* and rice is regarded as a classic model for studying the relationship between the pathogen and the host. In this study, we found a gene, *MoHG1*, regulating fungal development and virulence in *M. oryzae*. The ∆*Mohg1* mutants showed more sensitivity to cell wall integrity stressors and their cell wall is more easily degraded by enzymes. Moreover, a decreased content of chitin but higher contents of arabinose, sorbitol, lactose, rhamnose, and xylitol were found in the ∆*Mohg1* mutant. Combined with transcriptomic results, many genes in MAPK and sugar metabolism pathways are significantly regulated in the ∆*Mohg1* mutant. A hexokinase gene, *MGG_00623* was downregulated in ∆*Mohg1,* according to transcriptome results. We overexpressed *MGG_00623* in a ∆*Mohg1* mutant. The results showed that fungal growth and chitin contents in *MGG_00623*-overexpressing strains were restored significantly compared to the ∆*Mohg1* mutant. Furthermore, MoHG1 could interact with MGG_00623 directly through the yeast two-hybrid and BiFC. Overall, these results suggest that MoHG1 coordinating with hexokinase regulates fungal development and virulence by affecting chitin contents and cell wall integrity in *M. oryzae,* which provides a reference for studying the functions of *MoHG1*-like genes.

## 1. Introduction

*Magnaporthe oryzae* causes rice blast disease, which threatens global rice production. The pathogen can infect various grass hosts besides rice [1,2]. Genomic analyses revealed that there might be 10 distinct *M. oryzae* lineages and isolates from each lineage only infect a single host [3]. Recently, the emergence of *M. oryzae Triticum* (*MoT*) has caused serious losses in wheat production. The newest finding underlined that *MoT* was generated from the cross progeny of *Eleusine*- and *Urochloa*-infecting isolates and underwent a series of matings with a small number of individuals from three additional host-specialized populations [4]. However, phylogenetic analyses indicated a distant relationship between rice- and wheat-infecting populations. Our previous results also found a stronger selective sweep in the rice-infecting population than in the wheat-infecting population [5]. Thus, explorations of host selection mechanisms by *M. oryzae* favor decreasing the pathogen risk.

The fungal cell wall is composed of two layers. The inner layer has a cross-linked chitin–glucan matrix, and the outer layer possesses mannosylated proteins [6]. Chitin is a core component of the fungal cell wall, and UDP-N-acetylglucosamine is the substrate for chitin synthesis. Chitin is also a key elicitor to trigger plant immune response. Chitin modification in pathogens and recognition by the host are determinants of disease occurrence [7,8]. There are two effectors, MoChia1 and Slp1, which influence chitin modification and infection in *M. oryzae* [9,10]. Moreover, melanin is also found in the cell wall of numerous fungal pathogens, which mainly reinforces cell structure and maintains the pressure from appressorium [11,12]. So far, diverse chitin and melanin synthesis enzymes have been identified, most of which are essential for fungal development and infectious processes [7].

Carbon metabolism is essential for fungal cell wall development. Monosaccharides with glucosyl residues mainly take up 75% of monosaccharides of cell walls. Other monosaccharides are mannose (14%), N-acetylglucosamine (7%), galactose (2%), and traces of arabinose and xylose in *M. oryzae* [13]. The carbon catabolite repression (CCR) system is widely conserved in filamentous fungi and favors fungi to preferentially utilize a favorable carbon source [14]. In *M. oryzae*, Tps1 is responsible for glucose-6-phosphate sensing and triggers CCR via the inactivation of Nmr1-3 [15,16]. The CCR transcription factor MoCreA has been identified, which regulates the utilization of different carbon sources and gene expressions [17].

Many proteins on fungal cell walls preferentially perceive various cues from a host and regulate fungal infection. G-protein-coupled receptors Pth11 and WISH, hydrophobin proteins Mpg1 and Mhp1, transmembrane mucin Msb2, and membrane sensor MoSho1 were identified in *M. oryzae* [18,19,20,21]. These sensor proteins can transduce the signals for appressorium development in the of *M. oryzae*. Fungal cell wall integrity (CWI) proteins are essential for cell viability, morphogenesis, and pathogenesis. In *M. oryzae*, there are three mitogen-activated protein (MAP) cascade kinases responding to signal transduction, including MoMck1 (MAPKKK), MoMkk1 (MAPKK), and MoMps1 (MAPK) [22]. Diverse signal pathways identified have crosstalk with CWI, suggesting the central role of CWI in fungal growth and pathogenicity [23,24,25,26].

We previously found a gene encoding a hypothetical protein with a signal peptide and transmembrane domain by comparative genomics analysis, which might be involved in fungal virulence, but the functions of this gene were unknown. In this study, the gene-deletion mutants displayed compromised hypha growth, and the gene was named *MoHG1* (hypha growth gene in *M. oryzae*). *MoHG1* positively regulates fungal development and infection by maintaining CWI. Moreover, the decreased content of chitin was found in the ∆*Mohg1* mutant. Combined with transcriptomic results, many genes in MAPK and sugar metabolism pathways are significantly regulated in the ∆*Mohg1* mutant. A hexokinase gene, *MGG_00623* was downregulated in ∆*Mohg1* according to transcriptome results. We overexpressed *MGG_00623* in a ∆*Mohg1* mutant. The results showed that fungal growth and chitin contents in *MGG_00623*-overexpressing strains were restored significantly compared to the ∆*Mohg1* mutant. Furthermore, MoHG1 could interact with MGG_00623 directly through yeast two-hybrid and BiFC. These results provide valuable references for the genes with similar structures in *M. oryzae.*

## 2. Materials and Methods

### 2.1. Phylogenetic Analyses

The homolog sequences of *MoHG1* were downloaded from different rice- or non-rice-infecting isolates in the NCBI database. FastTree software (Version 2.1.10) was used for phylogenetic analysis. The phylogenetic tree plot was generated using ChiPlot (https://www.chiplot.online, accessed on 16 August 2024).

### 2.2. Culture Conditions

Wild-type *M. oryzae* YN125 and mutant strains conserved at Yunnan Agricultural University were cultured on a potato sucrose agar (PSA) medium for growth. Complete medium (CM) and minimum medium (MM) were used for growth assays with different stressor treatments. Oatmeal medium (OM) was used for sporulation. For quantitative analyses of spore production, germ tube, and appressorium formations, the conidia were harvested from OM and then filtered through two layers of Miracloth (Calbiochem, San Diego, CA, USA). The spore suspensions were resuspended twice in sterile water. Spore production in a given colony was counted using a hemocytometer. The spore suspension was dropped on a microscope cover glass (Fisher Scientific, 12545100, Waltham, MA, USA), and the rates of germ tube and appressorium formations were recorded using a microscope. The experiments were performed in three biological repeats.

### 2.3. Infection Assays

The susceptible rice variety Lijiangxintuanheigu (LTH) was used for fungal inoculation. A spore suspension (1 × 10^5^ spores/mL) was foliar-sprayed on 21-day-old rice seedlings. The inoculated seedlings were incubated in the dark for 24 h at 28 °C and transferred to the greenhouse for 6 days. At 7 days post-inoculation, the disease index was assessed. Fifteen seedlings were used for inoculation of each isolate, and there were three independent inoculation experiments. A disease score of 0–5 was assigned according to the lesion type. The disease index was calculated using the following formula.

$$Disease\;index=\frac{\sum (disease\;score\times number\;of\;diseased\;leaves)}{Total\;number\;of\;investigate\;leaves\times 5}\times 100$$

### 2.4. Protoplast Release Assay

The fungi were grown in liquid yeast extraction and glucose media (YEG) for 2 days and then harvested. The dried mycelia were transferred into a lysis enzyme solution for 1 h. The released protoplast solution was dropped on a hemocytometer and the number of protoplasts was counted using a microscope [23,24,25,26].

### 2.5. Vector Construction and Transformation

The vector pCX62 was used for *MoHG1* gene deletion, and the 1000–2000 bp sequences upstream and downstream of the *MoHG1*-coding sequences were inserted into two flanking regions of the maker gene (hygromycin phosphotransferase, HPT), respectively. The constructed deletion vector was transformed into protoplasts of YN125 using the PEG–CaCl_2_-mediated method. The positive deleted transformants were confirmed using PCR with specific primers (Table S1). The native promoter and coding sequences of *MoHG1* were inserted into a pYF11 vector carrying a green fluorescent gene and transformed into YN125 for MoHG1-GFP observation. For *MGG_00623*-overexpressing strain construction, coding sequences of *MGG_00623* were inserted into pYF11 with the continuous expressing promoter *RP27* and transformed into a ∆*Mohg1* mutant. The overexpression of *MGG_00623* was confirmed via qRT-PCR.

### 2.6. Carbohydrate Content Assay

Fungal strains were grown in liquid YEG for 2 days. The mycelia of WT and ∆*Mohg1* were dried using vacuum freezing and then ground into powder, respectively. 30 mg of the powder from each sample was diluted into a 500 μL solution containing methanol/isopropanol/water (3:3:2 *v*/*v*/*v*), vortexed for 3 min, and subjected to ultrasound for 30 min. The extraction was centrifuged at 4 °C and 14,000 r/min for 3 min. 20 μL suspensions were transferred to a new tube and added to 20 μL ribitol (100 μg/mL) as the internal standard. For the derivatization treatment, the mixture was dried using nitrogen gas and added to a 100 μL solution of methoxyamine hydrochloride in pyridine (15 mg/mL) for incubation at 37 °C for 2 h. Then, 100 μL of BSTFA was added to the mixture and maintained at 37 °C for 30 min.

An Agilent 7890 (Santa Clara, CA, USA) gas chromatograph coupled with a 5975C mass spectrometer with a DB-5MS column (30 m length × 0.25 mm i.d. × 0.25 μm film thickness, J&W Scientific, Folsom, CA, USA) was used for sugar detection. Helium was used as the carrier gas at a flow rate of 1 mL/min. Injections were performed in the spitless mode, and the injected volume was 2 μL. The oven temperature was maintained at 70 °C for 1 min, raised to 112 °C at 30 °C/min and maintained for 3 min, raised to 175 °C at 15 °C/min and maintained for 1 min, raised to 190 °C at 3 °C/min and maintained for 2 min, then raised to 240 °C at 35 °C/min, and finally raised to 280 °C at 10 °C/min and maintained at this temperature for 2.5 min. All samples were analyzed in the selective ion-monitoring mode. The ion source and transfer line temperatures were 230 °C and 240 °C, respectively.

The standard curve for each sugar was generated using the different concentrations of standard sugar solutions and corresponding mass spectrometer peak data. The sugar content was calculated using the following equation: content (mg/g) = c × V1 × V2 ÷ V3 ÷ m ÷ 1000000 (c is the concentration generated by the standard curve according to the peak area of each sample; V1 is the constant volume; V2 is the extraction volume; V3 is the collected suspension volume; m is the sample weight).

### 2.7. RNA Isolation and Quantitative Real-Time (qRT)-PCR

The RNA isolations from fungi and rice leaves were performed using the UNlQ-10 Column TRIzol Total RNA Isolation Kit (Sangon Biotech, B511321, Shanghai, China). PrimeScript IV 1st strand cDNA Synthesis Mix (Takara, 6215A, Shiga, Japan) was used for cDNA synthesis. The primers used for qRT-PCR are listed in Table S1. The *M. oryzae* actin gene *MGG_03982* and the rice actin gene were used as the endogenous controls for normalization, respectively. The relative expressions of target genes were calculated using the 2^−∆∆Ct^ method.

### 2.8. RNA Sequencing

The RNA sequencing methods for WT and ∆*Mohg1* isolates were performed based on the previous protocols [27]. Fungal strains were grown in liquid YEG for 2 days and then the mycelia were collected for RNA isolation. Sequencing libraries were constructed using the Illumina TruseqTM RNA sample prep kit (San Diego, CA, USA) and sequenced on the Illumina Novaseq 6000 platform (San Diego, CA, USA). The raw data were uploaded to NCBI SRA under the BioProject number PRJNA1076595. Clean data were assembled using Cufflinks (Version 2.2.1) based on the reads mapped to the *M. oryzae* reference genome (https://www.ncbi.nlm.nih.gov/assembly/GCF_000002495.2, accessed on 16 August 2024), while unmapped reads were annotated with sequence alignment. The read count of each gene was generated by RSEM software (Version 1.3.3), DESeq2 (Version 1.10.1) was used to identify differentially expressed genes (DEGs), and the DEGs were filtered by |log_2_FC| ≥ 1, with a Padjust value of <0.05. The GO and KEGG analyses were based on DAVID (https://david.ncifcrf.gov/home.jsp, accessed on 16 August 2024).

### 2.9. Cell Wall Staining and Chitin Content Assays

Calcofluor white (CFW) was used for fungal cell wall staining according to the methodology in a previous paper with a slight modification [23,24,25,26]. WT and ∆*Mohg1* were grown in liquid YEG for 4 days and washed using ddH_2_O. The dried mycelia were soaked in 10 μg/mL CFW solution for 5 min and rinsed with ddH_2_O to wash off the dye. The cell wall staining was observed using a fluorescence microscope.

The chitin content assays for WT and ∆*Mohg1* were conducted according to a previously published method [28]. WT and ∆*Mohg1* were grown in liquid YEG for 4 days and washed using ddH_2_O. The chitin content was determined by measuring the amount of glucosamine released after the hydrolysis of the cell walls.

### 2.10. Yeast Two-Hybrid and BiFC Assays

For yeast two-hybrid, the CDS fragment of *MoHG1* was inserted into prey plasmid pPR3-N, while the CDS fragment of the *MGG_00623* was cloned into bait plasmid pBT3-STE. The constructed vectors were co-transformed into competent cells of yeast NMY51 and incubated on a synthetic medium lacking leucine and tryptophan (SD–Leu–Trp) then were further transferred to a synthetic medium lacking leucine, tryptophan, adenine, and histidine (SD–Leu–Trp–Ade–His). For BiFC observation, the CDS regions of *MGG_00623* and *MoHG1* were cloned into pSm35s-nYFP and pSm35s-cYFP vectors, respectively. The pSm35s::nYFP::MGG_00623 and pSm35s::MoHG1::cYFP were co-inoculated into tobacco leaves through agrobacterium-mediated transformation and incubated for 2 days. The YFP was observed under a laser laser-scanning confocal microscope.

## 3. Results

### 3.1. MoHG1 Influence the Fungal Development

**Figure 1:**
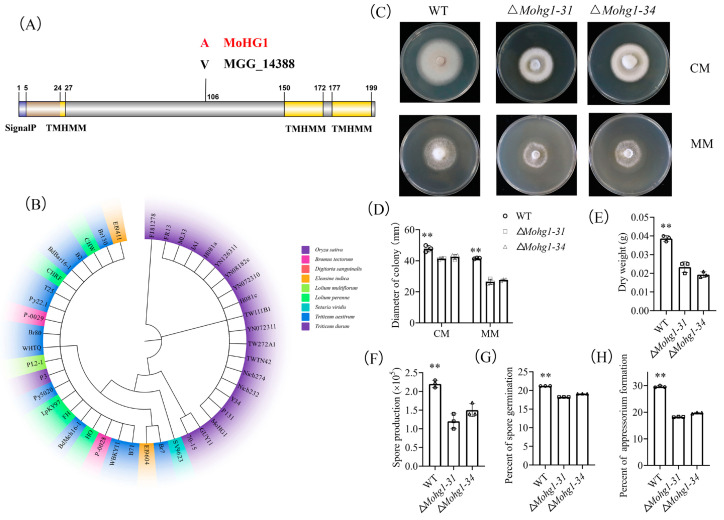
*MoHG1* influences the fungal development. (**A**) The amino acid sequence alignment between MoHG1 and MGG_14388. (**B**) Phylogenetic analyses of MoHG1 homologous sequences in different host-infecting strains. (**C**) Colonic phenotypes of ∆*Mohg1* at CM and MM plates. (**D**–**H**) The diameter of the colony, dry weight, spore production, spore germination, and appressorium formation. Each experiment was conducted with 3 biological repeats and statistically significant differences were calculated by Student’s *t*-test, ** *p* < 0.01. Error bars represent the means ± SD.

MoHG1 encodes 201 amino acids with a 24-amino acid signal peptide and possesses 3 transmembrane domains. There is one nonsynonymous substitution at the 106th amino acid locus between MoHG1 and MGG_14388 in the 70–15 reference strain genome (Figure 1A). We aligned the sequences of *MoHG1* in the genomes of rice and non-rice lineage isolates and found that the sequences in rice lineage were generally clustered together while the sequences in non-rice lineages belonged to another subgroup (Figure 1B).

Two ∆*Mohg1* mutants were screened using PCR (Figure S1), and the hyphal growth of both ∆*Mohg1* mutants was compromised when they were grown on the CM and MM plates compared with the wild type. Moreover, spore production, germination, and appressorium formation were also decreased in the ∆*Mohg1* mutants (Figure 1C–H). These results indicate that *MoHG1* affects fungal development.

### 3.2. MoHG1 Regulates Fungal Cell Wall Integrity in M. oryzae

**Figure 2:**
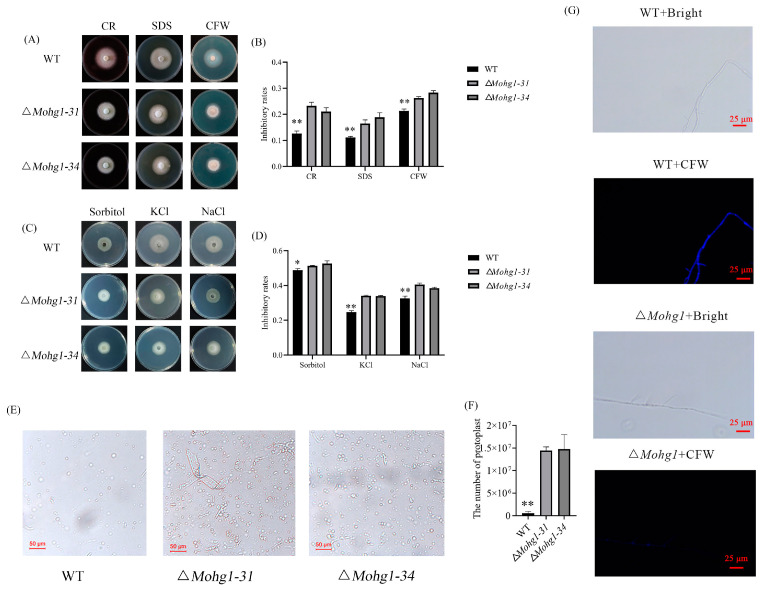
*MoHG1* regulates fungal cell wall integrity in *M. oryzae*. (**A**,**B**) ∆*Mohg1* mutants show more sensitivity to CR (600 μg/mL), SDS (100 μg/mL), and CFW (4 μg/mL). (**C**,**D**) ∆*Mohg1* mutants show more sensitivity to sorbitol (1 mol/L), KCl (0.7 mol/L), and NaCl (0.7 mol/L). (**E**,**F**) The released protoplast in WT and ∆*Mohg1* mutants. Each experiment was conducted with 3 biological repeats and statistically significant differences were calculated by Student’s *t*-test, * *p* < 0.05, ** *p* < 0.01. Error bars represent the means ± SD. (**G**) The fungal cell wall staining in ∆*Mohg1* and WT.

Congo red (CR), sodium dodecyl sulfate (SDS), and calcofluor white (CFW) are often used to assess fungal cell wall integrity. *Mohg1* mutants were more sensitive to CR, SDS, and CFW than WT (Figure 2A,B). Similarly, osmotic stressors such as sorbitol, KCl, and NaCl show stronger inhibition in ∆*Mohg1* mutants (Figure 2C,D). Moreover, fungal mycelia were treated with cell wall-degrading enzymes. The results showed more protoplasts were released by ∆*Mohg1* mutants than WT at 1 h post-treatment (Figure 2E,F). The fungal cell wall of each strain was stained by CFW, and there was a stronger fluorescence signal observed in WT than in the ∆*Mohg1* (Figure 2G). These results indicate that fungal cell wall integrity was disrupted in ∆*Mohg1* mutants, and MoHG1 plays an important role in maintaining CWI.

### 3.3. MoHG1 Plays an Essential Role in Pathogenicity

**Figure 3:**
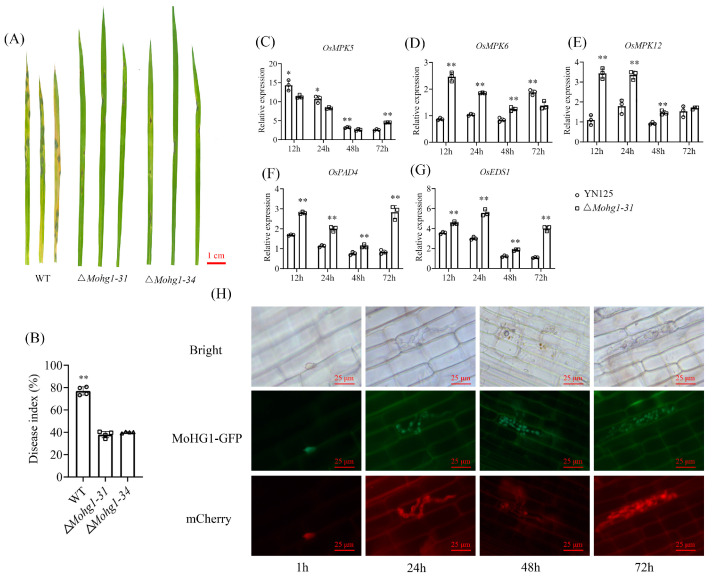
*MoHG1* plays an essential role in pathogenicity. (**A**,**B**) The pathogenicity of ∆*Mohg1* mutants on rice. (**C**–**G**) The expressions of basal defense genes in rice inoculated by ∆*Mohg1* and WT. (**H**) The fluorescent signal of MoHG1-GFP in *M. oryzae* during infection. Each experiment was conducted with 3 biological repeats and statistically significant differences were calculated by Student’s *t*-test, * *p* < 0.05, ** *p* < 0.01. Error bars represent the means ± SD.

To investigate the role of *MoHG1* in pathogenesis, spore suspensions of WT and ∆*Mohg1* mutants were sprayed onto rice seedlings. At day 7 post-inoculation, typically infectious lesions were formed by WT, while a smaller number of lesions were caused by the ∆*Mohg1* mutants (Figure 3A). Pathogenicity assay showed that the disease index of WT was much higher than that of the mutants (Figure 3B). Moreover, some basal defense genes such as *OsMPK5*, *OsMPK6*, *OsMPK12*, *OsPAD4,* and *OsEDS1* were upregulated in the rice inoculated with ∆*Mohg1-31* (Figure 3C–G). The fragments containing *MoHG1-GFP* with its native promoter were transferred into an mCherry fluorescent protein tagged strain YN125. The fluorescent signal of MoHG1-GFP was observed in spore and infectious mycelia during infection (Figure 3H). These results suggest that the expressions of MoHG1 can be induced and affect fungal pathogenicity during infection.

### 3.4. Sugar and MAPK Pathways Are Regulated in ∆Mohg1 by Transcriptome Analysis

**Figure 4:**
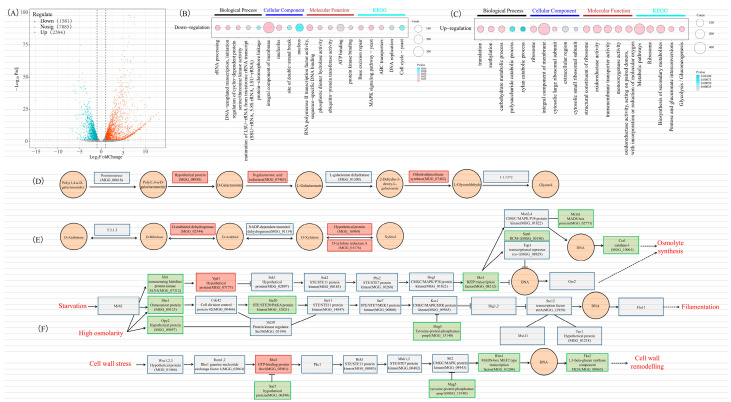
Transcriptome analysis between WT and ∆*Mohg1*. (**A**) The volcano plot of DEGs in ∆*Mohg1*. GO enrichment and KEGG pathway analysis of downregulated (**B**) and upregulated (**C**) genes in ∆*Mohg1*. The DEGs involving glycerol synthesis (**D**), synthesis pentose (**E**), and MAPK pathway (**F**) in *M. oryzae*. The box background in green or red means the decrease or increase of gene expression according to the transcriptome, respectively.

Transcriptomic analysis was performed to explore the expression patterns characterized by *MoHG1*. There were 2394 upregulated genes and 1581 downregulated genes in the ∆*Mohg1* mutant compared with WT (Figure 4A). Based on the GO and KEGG results, the downregulated genes were mainly enriched in RNA transcription, an integral component of the membrane, nucleolus, MAPK signaling pathway, and cell cycle (Figure 4B). Meanwhile, the upregulated genes were related to translation, methylation, the carbohydrate metabolic process, ribosome, pentose, and glucuronate interconversion-related biological processes in the ∆*Mohg1* mutant (Figure 4C). Based on the pentose and glucuronate interconversion pathway, there were many upregulated genes involved in the glycerol and xylitol metabolism (Figure 4D,E). Moreover, there were many downregulated genes in the MAPK pathway in the ∆*Mohg1* mutant (Figure 4F). Thus, transcriptome analysis indicated that *MoHG1* could regulate sugar and MAPK pathways to affect fungal growth.

### 3.5. MoHG1 Influences the Contents of Some Carbohydrates

**Figure 5:**
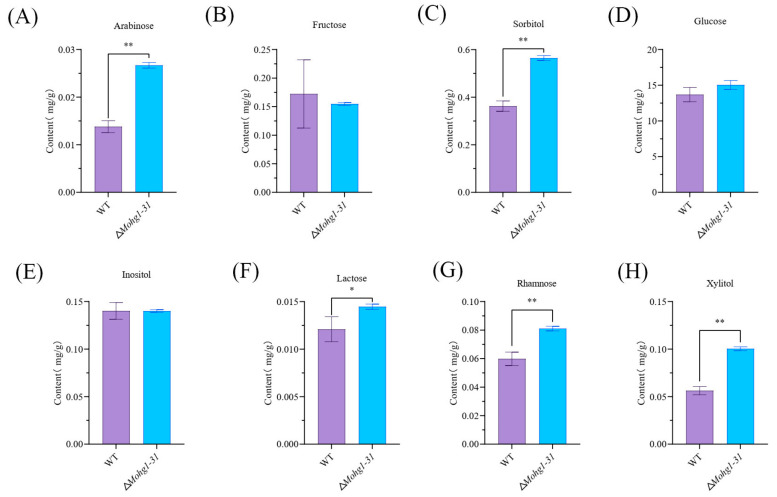
The carbohydrate contents in ∆*Mohg1*. (**A**–**H**) The content of 8 carbohydrates. Each experiment was conducted with 3 biological repeats and statistically significant differences were calculated by Student’s *t*-test, * *p* < 0.05, ** *p* < 0.01. Error bars represent the means ± SD.

According to the KEGG analyses, we found carbohydrate metabolism-related genes were upregulated in the ∆*Mohg1* mutant. Thus, the contents of carbohydrates, including arabinose, fructose, sorbitol, glucose, inositol, lactose, rhamnose, and xylitol were measured. The results showed that the contents of arabinose, sorbitol, lactose, rhamnose, and xylitol were significantly higher in the ∆*Mohg1* mutant than in WT (Figure 5A–H). The results show that *MoHG1* influences the contents of some carbohydrates to regulate fungal growth in *M. oryzae*.

### 3.6. Overexpressing of MGG_00623 in ∆Mohg1 Mutant Restored the Fungal Growth and Chitin Contents

**Figure 6:**
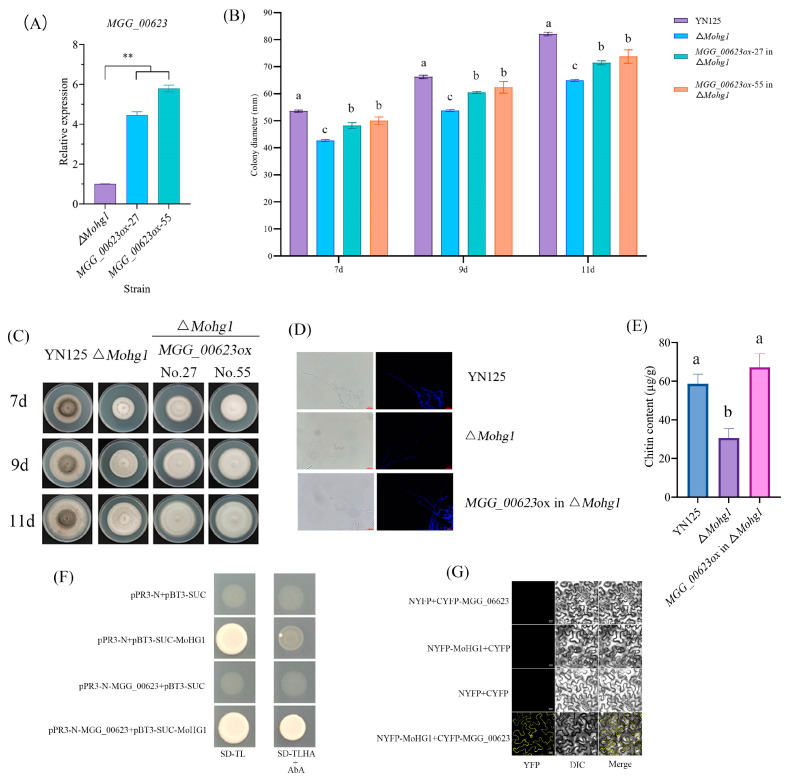
Overexpressing of *MGG_00623* in the ∆*Mohg1* mutant restored the fungal growth and chitin contents. (**A**) The relative expressions of *MGG_00623* in ∆*Mohg1*. Each experiment was conducted with 3 biological repeats and statistically significant differences were calculated by Student’s *t*-test, ** *p* < 0.01. (**B**,**C**) Overexpression of *MGG_00623* in ∆*Mohg1* partially restored the fungal growth. Each experiment was conducted in 3 biological repeats. The different letters above each bar graph indicate significant differences (*p* < 0.05) calculated by ANOVA and Duncan’s test. Error bars represent the means ± SD. (**D**,**E**) Overexpression of *MGG_00623* in ∆*Mohg1* restored CWI staining and chitin contents. The scale bar represents 25 μm. The different letters above each bar graph indicate significant differences (*p* < 0.05) calculated by ANOVA and Duncan’s test. Error bars represent the means ± SD. (**F**) Yeast two-hybrid and (**G**) BiFC assays show MoHG1 interacts with MGG_00623. The scale bar represents 20 μm.

Transcriptomic analysis indicated that *MoHG1* could affect cell wall integrity and carbohydrate metabolism. Coincidently, the contents of some carbohydrates and chitin were also disrupted in ∆*Mohg1* compared with WT (Figure 2G and Figure 5). For the downregulated genes in ∆*Mohg1*, *MGG_00623* encodes a hexokinase, which may be associated with carbohydrates and chitin metabolism. Thus, *MGG_00623* was overexpressed in ∆*Mohg1* mutants and two overexpression strains were obtained (Figure 6A). We found that the colony diameters of overexpressing strains were greater than ∆*Mohg1* (Figure 6B,C). Moreover, the CFW staining and chitin contents were restored significantly in overexpressing strains, suggesting the overexpressing of *MGG_00623* could promote the CWI (Figure 6D,E). We also found that MoHG1 could interact with MGG_00623 directly through yeast two-hybrid and BiFC (Figure 6F,G). Thus, our results showed that MoHG1 could interact with MGG_00623 and the overexpressing of *MGG_00623* in the ∆*Mohg1* mutant could restore the fungal growth and chitin contents.

## 4. Discussion

### 4.1. MoHG1 Is Required for Development and Pathogenicity of M. oryzae

In this study, we have identified a gene, *MoHG1,* involved in fungal development and pathogenicity in *M. oryzae*. *MoHG1* in the *M. oryzae* Oryza pathotype is more conserved than in other pathotypes, indicating that this gene might be associated with the differentiation of the Oryza pathotype in *M. oryzae* populations (Figure 1B). *MoHG1* possesses typical signal peptide and transmembrane domains, but the protein could not be secreted into the extracellular space and cytoplasm in rice under microscope observation (Figure 1A and Figure 3H). Moreover, previous secretome results did not identify this protein during the infection of *M. oryzae* [29]. A similar structural protein, PRO41, harboring a signal sequence and three transmembrane domains, was identified in *Sordaria macrospora*, which is located in the endoplasmic reticulum (ER) membrane and is essential for sexual development [30]. These results suggested that MoHG1 affected pathogenicity by regulating fungal development, not by being secreted into rice cells to disrupt host defense.

∆*Mohg1* mutants display compromised fungal development and pathogenicity compared with the wild type (Figure 1C–H and Figure 3A). These results suggest that MoHG1 plays an important role in fungal development and infection. Based on transcriptome analysis (Table S2), many transcription factor (TF) genes were downregulated in the ∆*Mohg1* mutant. *Con7* regulates appressorium formation [31]. Homeobox TF family genes, such as *MoHOX2* and *MoHOX4*, are essential for conidiogenesis [32]. *Com1*, *Moatf1*, and *MoMcm1* are required for fungal development and virulence [33,34,35]. Thus, *MoHG1* might affect the expression of some TF genes to regulate downstream genes to influence development and pathogenicity in *M. oryzae*.

Many innate immune-related genes were upregulated in rice inoculated with the ∆*Mohg1* mutant (Figure 3C). The MAPK cascade genes *OsMPK6* and *OsMPK12* (*OsBWMK1*) were upregulated, which were associated with the *WRKY45*- and *OsWRKY33*-mediated SA signaling pathways, respectively [36]. Furthermore, *OsPAD4* and *OsEDS1* involved in the SA pathway were also induced by the ∆*Mohg1* mutant. These results suggested that the activation of an SA pathway in rice enhances the resistance with ∆*Mohg1*. Effectors secreted by pathogens that interfere with the SA pathway have been identified. In *M. oryzae*, two nuclear effector gene deletion mutants, ∆*Mohtr1* and ∆*Mohtr2*, also induce expressions of SA-related genes when they were inoculated [37]. Interestingly, many genes encoding cell death-inducing proteins (CDIPs) [38,39] were upregulated in the ∆*Mohg1* mutant (Table S2) and cell death is regarded as an immunity response to pathogens. However, whether a ∆*Mohg1* mutant could secret more cell death-inducing proteins into rice cells and trigger SA-mediated immunity needs further research.

### 4.2. MoHG1 Regulates Signal Sensors and Cell Wall Integrity in M. oryzae

In *M. oryzae*, signal transduction from the cell surface into the cell through a series of phosphorylation cascades, such as the cyclic AMP-dependent protein kinase A (cAMP/PKA) signaling pathway [40], the Pmk1 MAPK pathway [41,42], and the CWI pathway, have been well identified [22,43]. The crosstalk among these pathways can coordinate them with each other to regulate fungal behavior. According to the KEGG results, many downregulated genes were enriched in the MAPK signaling pathway (Figure 4B). We found that the expressions of four sensor genes, *MoSho1*, *MoOpy2*, *Sln1,* and *PTH11* were downregulated in the ∆*Mohg1* mutant (Table S2). MoSho1 functions by recognizing rice leaf waxes for Pmk1 activation and appressorium formation [21]. MoOpy2 participates in the *Osm1* MAPK pathway and the Mps1 MAPK pathway to regulate fungal development, pathogenicity, and autophagy [44]. Sln1 is responsible for sensing the turgor threshold to affect penetration into rice leaves [45]. PTH11 as a G-protein-coupled receptor influences the MAPK Pmk1 and cAMP/PKA pathways to regulate appressorium [18]. In summary, *MoHG1* influences the expressions of sensor genes to regulate signal transduction.

The ∆*Mohg1* mutants showed more sensitivity to CWI stressors and more easily released protoplasts under lysis enzyme treatment (Figure 2). The CWI pathway is essential to pathogen development and infection. The core components of the CWI pathway have been well identified, but *MoMck1*, *MoMkk1*, and *MoMps1* were not significantly regulated in the ∆*Mohg1* mutant. However, *MoSLN1*, *MoGln2*, and *Gel5* were reported to participate in CWI [13,46,47] and downregulated in the ∆*Mohg1* mutant. Thus, MoHG1 may affect the expressions of CWI-related genes to maintain fungal cell wall integrity.

### 4.3. MoHG1 Interacts with a Hexokinase to Mediate Carbohydrate and Chitin Metabolism

Pentose and glucuronate interconversion pathway and glycolysis/gluconeogenesis pathway-related genes were significantly enriched in the ∆*Mohg1* mutant (Figure 4C), and the contents of arabinose, sorbitol, lactose, rhamnose, and xylitol were also higher in ∆*Mohg1* (Figure 5A,C,F–H). Interestingly, we also found many genes involving lactose, arabitol, and xylitol metabolism upregulated in the ∆*Mohg1* mutant (Figure 4C–E). The glycolysis/gluconeogenesis pathway is responsible for the breakdown of glucose into pyruvate, which can be further converted into lactate by L-lactate dehydrogenase [48]. The ∆*Mohg1* mutant was cultured in a nutrition-rich medium, which could provide sufficient glucose for growth, and thus the content of glucose in the ∆*Mohg1* mutant is similar to that in WT. Arabinose and xylitol are the metabolites of the pentose catabolic pathway [49]. The expressions of the D-arabinitol dehydrogenase and D-xylulose reductase A were upregulated in the ∆*Mohg1* mutant, and were consistent with higher contents of arabinose and xylitol in ∆*Mohg1*. These results suggest that *MoHG1* negatively regulates some aspects of carbohydrate metabolism. However, the relationship between higher contents and growth defect regulated by *MoHG1* in *M. oryzae* needs further research.

Fungal chitin is required for growth and virulence in *M. oryzae*. We found that the cell wall integrity was disrupted, and the content of chitin was also decreased in ∆*Mohg1*. There are seven chitin synthase (CHS) genes identified in *M. oryzae* [50]. Based on transcriptome analysis, *CHS1* and *CHS3* were downregulated, while *CHS2* and *CHS7* were upregulated in ∆*Mohg1,* which could affect chitin content (Table S2). Based on transcriptome results, many genes were downregulated that were involved in carbohydrate and chitin metabolism. These results might indicate that *MoHG1* could be an upstream component to regulate fungal carbohydrate and chitin metabolism. For the downregulated genes, *MGG_00623* encodes a hexokinase-1, which is regarded as the first enzyme to catalyze the conversion of glucose into glucose-6-phosphate in the glycolysis pathway, while glucose-6-phosphate can be converted into fructose-6-phosphate as the primary material for chitin synthesis in the hexosamine pathway. Many studies have shown that hexokinase-mediated glucose utilization can positively affect chitin synthesis in insects [51,52,53]. Our results indicate that MGG_00623 could play a more important role in *MoHG1* mediating fungal growth and virulence via the sugar and chitin metabolism pathway. Moreover, MGG_00623 could interact with MoHG1 directly, which might remodel the MGG_00623 structure and enhance the hexokinase activity to increase the chitin production in *M. oryzae*. However, our results demonstrate that *MoHG1* affects fungal growth and virulence through regulating CWI and chitin content. The molecular mechanisms of interactions among MoHG1, CWI, and chitin synthesis-related proteins still require further research.

## Data Availability

The raw data were uploaded to NCBI SRA under the BioProject number PRJNA1076595. [SUB14224803] [https://submit.ncbi.nlm.nih.gov/subs/sra/SUB14224803/overview, accessed on 16 August 2024] [SUB14224803].

## Supplementary Materials

The following supporting information can be downloaded at: https://www.mdpi.com/article/10.3390/jof10090663/s1, Figure S1: The confirmation of ∆*Mohg1* mutants by PCR; Table S1: The primers used in this study; Table S2: The DEGs in ∆*Mohg1* compared with WT.
