# Supplementary figures and images for: MoHG1 Regulates Fungal Development and Virulence in Magnaporthe oryzae
Source: J Fungi (Basel). 2024 Sep 21;10(9):663. doi: 10.3390/jof10090663 (PMC11433375; doi:10.3390/jof10090663)

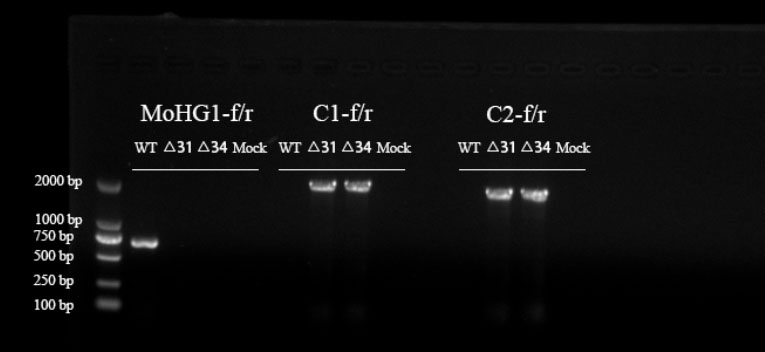

Supplement: Supplementary file 1 [file jof-10-00663-s001.zip › jof-3187329-supplementary/FigS1.jpg]
